# A dynamical systems model of progesterone receptor interactions with inflammation in human parturition

**DOI:** 10.1186/s12918-016-0320-1

**Published:** 2016-08-19

**Authors:** Douglas Brubaker, Alethea Barbaro, Mark R. Chance, Sam Mesiano

**Affiliations:** 1 Center for Proteomics and Bioinformatics, Case Western Reserve University, 11900 Euclid Avenue, Cleveland, 44106 OH USA; 2 11900 Euclid Avenue, Cleveland, 44106 OH USA; 3 Department of Reproductive Biology, Case Western Reserve University, 11900 Euclid Avenue, Cleveland, 44106 OH USA

**Keywords:** Myometrium, Progesterone receptor, Inflammation, Parturition, Dynamical systems

## Abstract

**Background:**

Progesterone promotes uterine relaxation and is essential for the maintenance of pregnancy. Withdrawal of progesterone activity and increased inflammation within the uterine tissues are key triggers for parturition. Progesterone actions in myometrial cells are mediated by two progesterone receptor (PR) isoforms, PR-A and PR-B, that function as ligand-activated transcription factors. PR-B mediates relaxatory actions of progesterone, in part, by decreasing myometrial cell responsiveness to pro-inflammatory stimuli. These same pro-inflammatory stimuli promote the expression of PR-A which inhibits the anti-inflammatory activity of PR-B. Competitive interaction between the progesterone receptors then augments myometrial responsiveness to pro-inflammatory stimuli. The interaction between PR-B transcriptional activity and inflammation in the pregnancy myometrium is examined using a dynamical systems model in which quiescence and labor are represented as phase-space equilibrium points. Our model shows that PR-B transcriptional activity and the inflammatory load determine the stability of the quiescent and laboring phenotypes. The model is tested using published transcriptome datasets describing the mRNA abundances in the myometrium before and after the onset of labor at term. Surrogate transcripts were selected to reflect PR-B transcriptional activity and inflammation status.

**Results:**

The model coupling PR-B activity and inflammation predicts contractile status (i.e., laboring or quiescent) with high precision and recall and outperforms uncoupled single and two-gene classifiers. Linear stability analysis shows that phase space bifurcations exist in our model that may reflect the phenotypic states of the pregnancy uterus. The model describes a possible tipping point for the transition of the quiescent to the contractile laboring phenotype.

**Conclusions:**

Our model describes the functional interaction between the PR-A:PR-B hypothesis and tissue level inflammation in the pregnancy uterus and is a first step in more sophisticated dynamical systems modeling of human partition. The model explains observed biochemical dynamics and as such will be useful for the development of a range of systems-based models using emerging data to predict preterm birth and identify strategies for its prevention.

## Background

Preterm birth (PTB) causes the majority of neonatal mortality and morbidity and is a major public health and socioeconomic problem worldwide [1, 2]. To prevent PTB, a clear understanding is needed of the hormonal interactions and signaling pathways that control the uterine contractile state. For most of pregnancy the myometrium (uterine muscle) is maintained in a relaxed and quiescent state to accommodate the growing conceptus. Parturition is initiated by a dramatic phenotypic transformation of the myometrium to the laboring state wherein it becomes the rhythmically contracting engine for birth. It is generally considered that the contractile state of the myometrium is controlled by the balance between the relaxatory influences of the steroid hormone progesterone and pro-labor stimuli, especially tissue-level inflammatory stimuli within the myometrium [6]. Progesterone is essential for the establishment and maintenance of pregnancy and its withdrawal is the principle trigger for parturition [3–7]. Multiple studies support the concept that parturition is associated with increased tissue-level inflammation within the myometrium, decidua, and cervix [8–10].

Actions of progesterone in myometrial cells are mediated by two progesterone receptor (PR) isoforms, designated PR-A and PR-B, that function as ligand activated transcription factors with PR-B exhibiting stronger transcriptional activity than PR-A. In vitro studies show that PR-A acts as a repressor of progesterone responsiveness by inhibiting the transcriptional activity of PR-B at certain promoters [11–13]. In most species progesterone withdrawal occurs by a decrease in circulating progesterone levels [14–18]. Human parturition occurs without systemic progesterone withdrawal, and instead is thought to involve decreased responsiveness of the myometrial cell to PR mediated progesterone actions resulting in a functional progesterone withdrawal [8, 19].

Previous studies have shown that most of human pregnancy, progesterone via PR-B promotes uterine quiescence, in part by inhibiting the responsiveness of myometrial cells to pro-inflammatory stimuli and preventing tissue level inflammation, and that functional progesterone withdrawal at parturition is caused by increased PR-A-mediated trans-repression of PR-B [8, 19, 20]. As pregnancy advances, the capacity for PR-B to mediate relaxatory and anti-inflammatory actions of progesterone on the pregnancy myometrium decreases due to increased repression by PR-A [20]. Interestingly, the amount and transrepressive activity of PR-A in myometrial cells is increased by pro-inflammatory stimuli suggesting a causal link between inflammation and PR-A-mediated functional progesterone withdrawal [21]. Thus, our working model for functional progesterone withdrawal in the control of human parturition posits that PR-B-mediated progesterone actions in the myometrium gradually decreases with advancing gestation in response to gradual increases in PR-A in response to increased inflammatory load. This mechanism is referred to as the PR-A:PR-B hypothesis for functional progesterone withdrawal [8, 20, 22].

Dynamical systems modeling uses fixed rules to describe the behavior of a system as its interacting components change with time. This framework has been used to examine the temporal activity of multiple biological systems including epidemics [23], predator-prey population interactions [24], chemical kinetics, protein phosphorylation, and cell signaling pathways [25, 26]. When the mechanism underlying the dynamics of a system is not well understood, a dynamical systems model can be useful for determining whether a particular a particular set of hypotheses that underly the model constitute a plausible mechanism by examining if the predictions of that model are borne out by the data. For all these reasons, dynamical systems are well suited for modeling the process of parturition where the myometrium undergoes a dramatic phenotypic bifurcation as it changes from the quiescent to laboring phenotype and the precise mechanism for this transformation is not yet known.

Herein we present a dynamical systems model consistent with the PR-A:PR-B hypothesis that links PR-B activity and inflammatory status in the myometrium at term. PR-B and inflammation were each modeled with a differential equation describing their activation and generation rates, their limiting behavior, and how they interact in association with the onset of labor. The model was robust when tested using published transcriptome datasets from quiescent and laboring myometrium and predicted contractile status (i.e, laboring or quiescent) with high precision using a novel classifier developed from the model. This simple model is a first step in producing patient specific pregnancy trajectories to predict the onset of labor and provides a framework to clinically assess women at risk of preterm birth.

## Methods

### Model definition

**Fig. 1:**
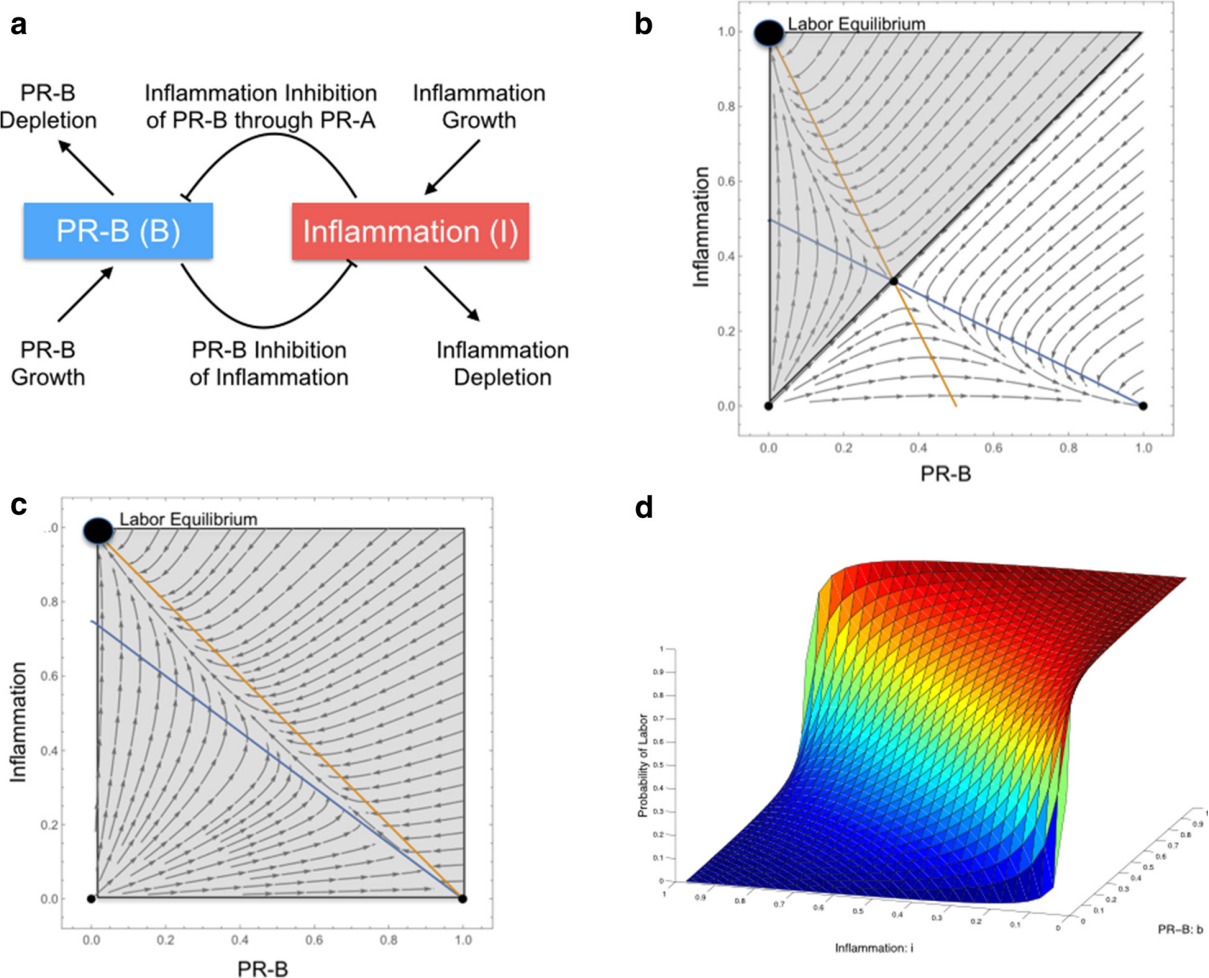
Model Definition and Properties. **a** Setup of the competitive interaction model between PR-B and inflammation where each variable has a growth term and acts to inhibit and deplete the other. **b** A setting of the phase space when *k*>*i* and *b*=0.5 where probability of labor is equal to 0.5 indicated by the shaded region, the basin of attraction, about the laboring equilibrium point. **c** A setting of the phase space when *k*=*i* where regardless of the value of *b* the probability of labor is equal to 1. The blue and orange lines are the null clines and correspond to the lines produced when we set $\frac {dB}{d\tau }=0$ and $\frac {dI}{d\tau }$=0. **d** The dependence of the probability of labor upon the parameter values *b* and *i* for a *k* fixed at 1 and the model used to make predictions

A host of experimental data has been collected which links PR-A, PR-B, and inflammatory drivers in the pregnancy uterus [7, 8, 20–22, 27, 32]. We translated the principles of the PR-A:PR-B hypothesis into equations which could be used to mathematically explore the dynamics and consistency of the biological hypothesis of how the progesterone receptors interact with inflammation during pregnancy. In essence, the PR-A:PR-B hypothesis describes a standard competitive interaction between the pro-pregnancy actions of PR-B and the pro-labor actions of PR-A where the activity of PR-A is related to the level of inflammation in the myometrium. As such, we chose to consider only PR-B and inflammation and incorporated the effects of PR-A into the inflammatory terms of the model (Fig. 1a).

Two coupled differential equations were used to model the change in transcriptionally active PR-B over time, $\frac {d\hat {B}}{dt}$, and the change of inflammation over time, $\frac {d\hat {I}}{dt}$, as a function of the growth and depletion of each parameter and interactions between parameters. The equations we propose to model the transcriptional activity of PR-B and inflammation status are

1 $$\begin{array}{@{}rcl@{}} \frac{d\hat{B}}{dt} &=& \hat{b}\hat{B}\left(1-\frac{\hat{B}}{B_{c}}\right)-k_{1}\hat{B}\hat{I},\\ \frac{d\hat{I}}{dt} &=& \hat{i}\hat{I}\left(1-\frac{\hat{I}}{I_{c}}\right)-k_{2}\hat{B}\hat{I}.  \end{array} $$

The growth of PR-B and inflammation is modeled by the terms $\hat {b}\hat {B}(1-\frac {\hat {B}}{B_{c}})$ and $\hat {i}\hat {I}(1-\frac {\hat {I}}{I_{c}})$ respectively. These terms imply that the levels of PR-B and inflammation increase in the presence of PR-B, $\hat {B}$, and inflammation, $\hat {I}$, at rates $\hat {B}$ and $\hat {I}$ respectively. The terms $(1-\frac {\hat {B}}{B_{c}})$ and $(1-\frac {\hat {I}}{I_{c}})$, impose a maximum or *critical* value on the level of PR-B and inflammation. At any given time, there is a finite level of PR-B induced activation of the transcriptional machinery. This critical level of PR-B is represented by the parameter *B*_*c*_. Analogously, there is some saturable level of inflammatory drivers active in a myometrial cell, represented by the parameter *I*_*c*_. The growth terms $\hat {b}\hat {B}$ and $\hat {i}\hat {I}$ in the equations for PR-B and inflammation will themselves increase in size as the amount of PR-B and inflammation increase, but in a way that is limited by the critical values for PR-B and inflammation. If $\hat {B} =B_{c}$ or $\hat {I} = I_{c}$, the limiting terms in parenthesis equal zero which causes the growth term to equal zero.

The depletion of PR-B and inflammation is modeled by the terms after the negative sign, namely $k_{1}\hat {B}\hat {I}$ and $k_{2}\hat {B}\hat {I}$. Qualitatively, this means that the rate of depletion of PR-B is the product of $\hat {B}$, $\hat {I}$, and a rate constant *k*_1_. The depletion of inflammation follows the same behavior with a different rate constant *k*_2_. The value of *k*_1_ accounts for the relative amount of PR-B to repress inflammation and *k*_2_ accounts for the relative impact of inflammation on PR-B activity. While we know that the phenomenon of PR-B repression of inflammation occurs, the exact mechanism for this repressive activity is not well understood. By allowing for *k*_1_ and *k*_2_ to take on different values relative to one another, we are able to explore multiple possible models for PR-B repression of inflammation.

### Model nondimensionalization and simplification

Nondimentionalization is a tool for simplifying our model whereby the six parameters in our model, $k_{1}, k_{2}, B_{c}, I_{c}, \hat {i},$ and $\hat {B}$, are replaced with three dimensionless constants (For full derivation of the dimensionless model see Appendix of derrivations). While this can make it difficult to pinpoint the influence of individual parameters on the system’s behavior, since we only have six parameters in our model, unpacking the influence of particular dimensionless constants is straightforward. The units for $\hat {I}$, $\hat {B}$, *B*_*c*_, and *I*_*c*_ are the amount of PR-B or inflammation present, similar to a concentration. Time *t* is given in weeks. The rate constants $\hat {I}$ and $\hat {B}$ are in units $\frac {1}{\text {weeks}}$ while the rate constants *k*_1_ and *k*_2_ are in units $\frac {1}{\text {concentration} * \text {weeks}}$. We define three dimensionless variables for our model, $B = \frac {\hat {B}}{B_{c}}$, $I = \frac {\hat {I}}{I_{c}}$, and *τ*=*tk*_1_*I*_*c*_. Substituting these for $\hat {B}$, $\hat {I}$, and *t* yields the model,

2 $$\begin{array}{@{}rcl@{}} \frac{dB}{d\tau} = bB(1-B)-BI, \quad \quad \frac{dI}{d\tau} = iI(1-I)-kBI, \end{array} $$

where $b = \frac {\hat {b}}{I_{c}k_{1}}, i = \frac {\hat {i}}{I_{c}k_{1}},$ and $k = \frac {B_{c}k_{2}}{I_{c}k_{1}}.$

### Determining parameter values from transcriptome data

We obtained data from two published studies which examined transcriptional changes in the myometrium (obtained at the time of cesarean section delivery) of women who were not in labor (NIL: closed and rigid cervix and no indication of uterine contractions) and in labor (IL: cervix dilated >4 cm and rhythmic contractions). One study [28] in which transcriptome analysis was performed by microarray technology (henceforth referred to as the microarray dataset or microarray data) comprised 3 myometrial samples from NIL women at term (>37 weeks gestation), 3 samples from IL women at term, and 3 samples from IL women undergoing preterm (<37 weeks gestation) cesarean section delivery. Some confusion has emerged since publication about whether one of the term IL samples was truly in labor. We excluded this sample for our analysis of this dataset and combined the preterm and term IL samples into one IL group. The other study [29] used RNA sequencing (henceforth referred to as the RNAseq dataset or RNAseq data) of myometrium from 5 NIL women and 5 IL women at term. Both datasets collected independent, non-paired samples, comprising a total cohort of 18 unique samples (10 IL, 8 NIL).

We infer the activity of PR-B and pro-inflammatory drivers with two PR-B responsive genes to serve as surrogates for PR-B, FOXO1A and FKBP5 [20, 30], and three pro-inflammatory genes to serve as surrogates for inflammation, IL- 1*β*, IL-6 and IL-8 [31]. Although there may be more genes associated with PR-B and inflammatory response in the myometrium, a fully curated gene set does not exist for inferring the full set of transcriptome changes induced by PR-B and inflammation. Therefore, we focussed on a targeted set of well studied myometrium specific genes for inferring the activity of PR-B and inflammation in the model. The normalized values of the data for these genes *N*_*j*_, are calculated using the equation, $N_{j} = \frac {G_{j}- m}{M-m},$ where *G*_*j*_ is the value of the gene for patient *j*, *M* is the maximum expression value for that gene across patients in the dataset, and *m* is the minimum value of that gene across patients in the dataset. One consequence of this normalization procedure is that the transcriptome data has been nondimensionalized enabling integration of the dimensionless trascriptome data with the dimensionless constants. In this formulation the values of the surrogate genes parameterize the dimensionless model for each patient.

The normalization equation bounds the values for the PR-B and inflammation surrogates from 0 to 1 and makes the natural choice of values for critical levels of PR-B and inflammation *B*_*c*_=*I*_*c*_=1. We then assign the values of *b* and *i* using the normalized dimensionless values for the PR-B and inflammatory surrogate genes respectively and the value of *k* corresponds to the strength of PR-B’s anti-inflammatory actions. The values of *b* and *i* are determined by the normalized intensity of one of the PR-B and one of the inflammatory surrogate genes from the mRNA expression studies [28, 29] and there are six possible combinations of values for *b* and *i* depending upon which inflammatory and PR-B surrogate genes are chosen. Thus, for a particular patient, the values of *b* and *i* are determined by the normalized expression intensity from the RNA-seq or microarray study for one pair PR-B and inflammatory surrogate genes.

### Calculating the probability of labor for each patient

Next, we quantify the behavior of *k* in order to apply our model to patient data. To do this, we have to derive the steady state solutions for our model. These solutions are the values of *B* and *I* which cause $\frac {dB}{d\tau }=\frac {dI}{d\tau }=0$ and correspond to a state where the system undergoes no change. There are four steady states, also known as *equilibrium points*. These occur when the ordered pair for PR-B and inflammation, (*B*^∗^,*I*^∗^), is equal to, (0, 0), (1, 0), (0, 1), and $\left (\frac {i(1-b)}{k-ib},\frac {b(k-i)}{k-ib}\right)=\left (\frac {i^{2}b(1-k)-ik(1-b)}{k(bi-k)}, \frac {kb(i-1)}{bi-k}\right)$. Of these, (0,0) is the trivial equilibrium point where neither PR-B nor inflammation is present, (1,0) is the *quiescent equilibrium* where PR-B is maximal and there is no inflammation, and (0,1) is the *laboring equilibrium* where there is no PR-B and inflammation is maximal. The quiescent equilibrium correspond to a PR-B dominant state and laboring equilibrium corresponds to an inflammatory dominant state. The fourth equilibrium point, the *intermediate equilibrium*, exists only for certain values of *b* and *i* between the quiescent and laboring equilibrium (For full derivation of equilibrium points see Appendix of derrivations).

Since the values of *b* and *i* are determined by PR-B and inflammatory surrogate genes scaled from 0 to 1, these terms are bounded to that interval. Furthermore, since *B* and *I* are bounded by *B*_*c*_=1 and *I*_*c*_=1, *B*^∗^ and *I*^∗^ are bounded to the square domain with vertices (0,0), (0,1), (1,1), and (1,0) and area 1. So, the intermediate equilibrium point, in both forms, should satisfy the constraints 0≤*B*^∗^≤1 and 0≤*I*^∗^≤1. By considering how this constraint impacts both forms of the intermediate equilibrium we can derive a set of constraints for the values of *k*. In order to allow for the full range of values of *i*, we find that *i* and *k* satisfy 0≤*i*<*k*≤1.

In the limit in the case where *i*=*k*, the intermediate equilibrium point equals the quiescent equilibrium point (1,0). If we visualize this state in *phase space* (Fig. 1b) we see that all the vectors point away from the quiescent equilibrium point toward the laboring equilibrium. In phase space, these vectors define *trajectories* that indicate how the system would evolve in time given a certain starting point. The set of vectors pointing toward the laboring equilibrium point is known as the *basin of attraction* for the laboring equilibrium point.

We compute a *probability of labor* equal to the area of the laboring equilibrium point’s basin of attraction divided by the area of the domain, which in our case is 1. The area of the basin changes as *k*, *i*, and *b* change. This probabilistic interpretation of a phase space is reasonable under the assumptions that all possible pairs of values of *B* and *I* in the domain occur with equal likelihood. While it is clear that in a physiological context there are values of *B* and *I* that are more likely at different time points in pregnancy, since this information is not readily available our assumption of uniformity enables us to compute the probabilities in the most agnostic way possible. Given more information about the distribution of *B* and *I* over the course of pregnancy, it could be possible to get a more precise estimate of the probability of labor, but such an extension is not possible at this t.

For example, in the case where *k*=*i* and *b* is fixed at 0.5, the probability of labor is equal to 1, the entire domain is the basin of attraction for the laboring equilibrium, which means that quiescence is impossible (Fig. 1c). In order to ensure that quiescence is a possibility, we set *k*=1 so that only one value of *i*, *i*=1, results in a probability of labor equal to 1, enabling us to explore the full range of values for *b* and *i*. With *k* fixed, we can plot the dependence of the probability of labor upon the values of *b* and *i* in a surface upon which all patients must fall given a value for PR-B and inflammatory activity. Thus, the model we apply to patient data is

3 $$\begin{array}{@{}rcl@{}} \frac{dB}{d\tau} = bB(1-B)-BI, \quad \quad \frac{dI}{d\tau} = iI(1-I)-BI. \end{array} $$

Each patient in the microarray and RNA-seq datasets has an expression value for each of the surrogate genes, FOXO1A, FKBP5, IL- 1*β*, IL-6 and IL-8. In the absence of proteomic data precisely quantifying the protein level activity of these genes in vivo the mRNA expression levels can be combined with the framework of our mathematical model to approximate the functional activity of these genes at the time of labor, i.e. using FOXO1A or FKBP5 for PR-B and IL- 1*β*, IL-6, or IL-8 for inflammation. We calculated a probability of labor for each patient in each dataset using all six possible combinations of surrogate genes (FOXO1A, IL- 1*β*), (FKBP5, IL- 1*β*), (FOXO1A, IL-6), (FKBP5, IL-6), (FOXO1A, IL-8), and (FKBP5, IL-8) where each surrogate was used to set the parameters *b* and *i* in our model. We will hereafter refer to a pair of surrogate genes as a *predictor*. A probability of labor was computed for each patient which corresponds to the size of the basin of attraction for the laboring equilibrium point given a predictor pair of surrogate genes for *b* and *i*.

### Classifier construction and assessment

After normalization within platform cohort, half of the IL and NIL samples from the total cohort of 18 were randomly selected as a training dataset to construct a classifier from five IL and four NIL samples. Probabilities of labor were calculated for each of the samples using a pair of predictor genes, one PR-B responsive and one inflammatory responsive, to value the parameters *b* and *i* in the model. Two nonparametric 95 % confidence intervals were computed for the the probabilities of labor for the NIL and IL samples. These intervals about the medians of the NIL and IL samples constituted the NIL and IL classifiers. If the intervals did not separate, then we discarded that classifier.

We assessed performance of successful classifiers by computing the probabilities of labor for the remaining nine samples using the same predictor genes. The nine samples in this test set of data were classified as IL, NIL, or a no-call depending on whether a sample’s probability fell into the training set confidence interval for IL, NIL, or somewhere in between. Precision and recall metrics were used to assess the classifier defined as

4 $$\begin{array}{@{}rcl@{}} \text{precision} &=& \frac{\text{correctly classified samples}}{\text{total classified samples}}\\ \text{recall} &=& \frac{\text{classified samples}}{\text{total samples}}.  \end{array} $$

This procedure of creating classifiers with training samples and predicting phenotypes for test samples was repeated for all 17,640 possible combinations of samples from the microarray and RNA-seq datasets. For each combination, classifier performance was assessed for probabilities of labor calculated from each of all six combinations of PR-B (FOXO1A, FKBP5) and inflammatory (IL- 1*β*, IL-6, and IL-8) responsive genes. Precision and recall metrics for each classifier were aggregated into an average F-score and the proportion of successful classifiers out of 17,640 possible classifiers were computed. These metrics assessed how the selection of samples for the training set influenced i) classifier performance sensitivity and ii) classifier construction sensitivity. The equations for F-score and classifier success rate (CSR) are

5 $$\begin{array}{@{}rcl@{}} \text{F-score} &=& \frac{2*\text{precision}*\text{recall}}{\text{precision}+\text{recall}}\\ \text{CSR} &=& \frac{\text{constructed classifiers}}{17,640}.  \end{array} $$

**Fig. 2:**
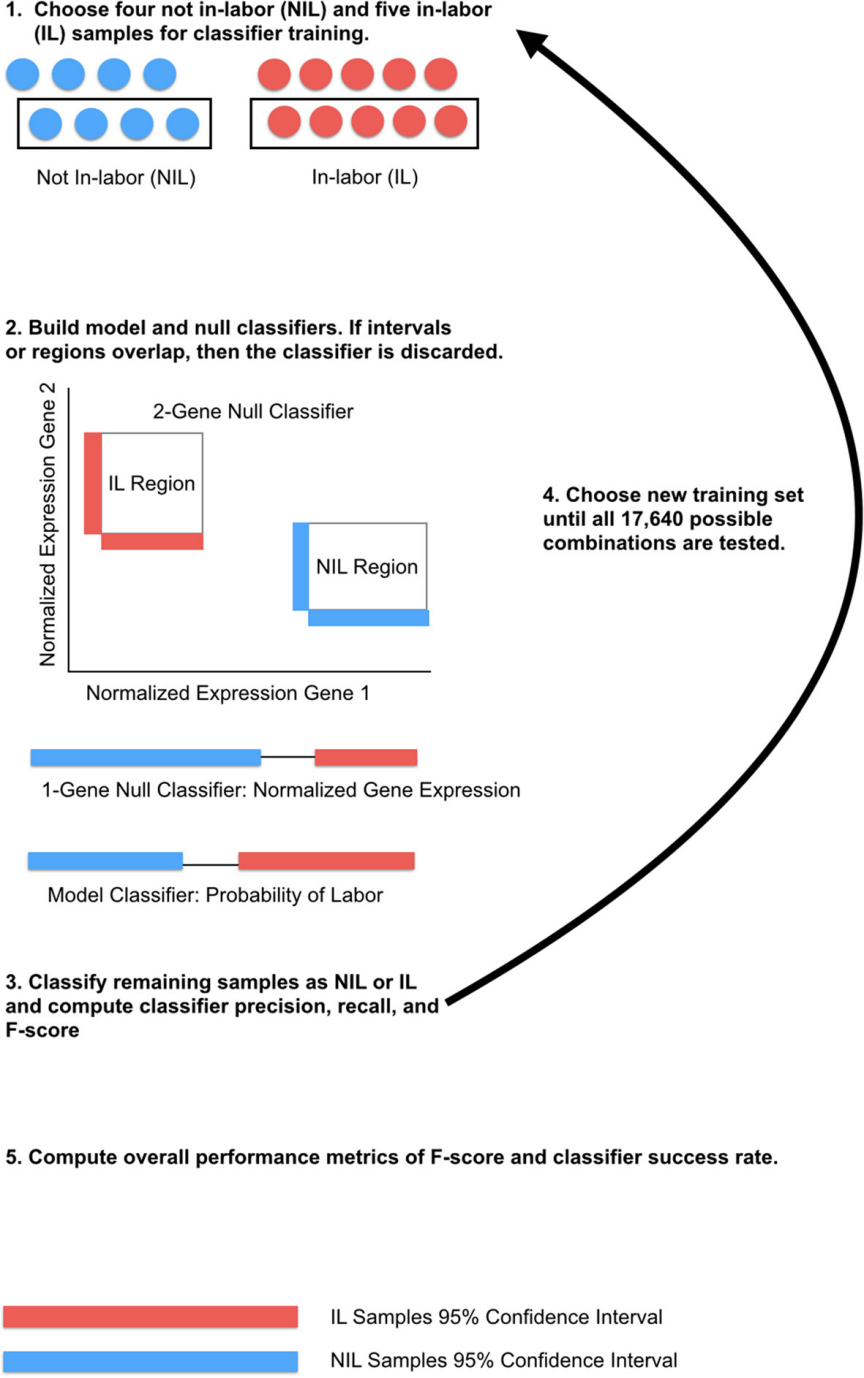
Classifier Construction and Assessment Workflow. A training set of half the IL and NIL samples was randomly sampled from our cohort of 18 myometrium samples. Probabilities of labor were computed for six combinations of predictor genes. 21 total classifiers were constructed for a particular combination of patients including five single gene null classifiers, 10 two-gene null classifiers, and six model classifiers. Performance of all classifiers was assessed by precision and recall metrics. All possible combinations of patient samples were assessed for classifier construction and overall performance metrics of the 21 classifiers were aggregated into average F-scores and classifier success rate (CSR)

These 105,840 model classifiers (17,640 training set combinations × 6 combinations of model predictor genes) were compared to two types of null classifiers. We constructed single and two-gene null classifiers for each of the 17,640 combinations of samples using the normalized expression values of the raw datasets. The single gene null classifiers were built by constructing 95 % nonparametric confidence intervals for the IL and NIL training samples on the normalized expression values for the individual genes. The two-gene null classifiers were constructed by defining a two dimensional confidence region for the two predictor genes for the NIL and IL training samples. Precision, recall, F-score, and CSR metrics were calculated for all 264,600 null classifiers (17,640 training set combinations x 15 one or two-gene null classifiers) and compared to the model classifiers to assess i) the relative performance of the single and two-gene null classifiers and ii) the performance of our model’s two-gene classifier to the null two-gene classifiers. A full workflow of this approach can be found in Fig. 2.

## Results and discussion

### Model results

The model has four equilibrium solutions, i.e. values for (*B,I*) pairs such that $\frac {dB}{d\tau } = \frac {dI}{d\tau } = 0$. At these values of *B* and *I*, namely (0, 0), (1, 0), (0, 1), and $\left (\frac {i(1-b)}{k-ib},\frac {b(k-i)}{k-ib}\right)$, the levels of PR-B and inflammation will remain constant. Each of these solutions corresponds to a physiological condition in the myometrium and the solutions can be *stable*, where the trajectories in the phase space in the neighborhood of the equilibrium point toward it, or *unstable*, where the trajectories in the neighborhood of the equilibrium point away from it.

The values of the parameters in the model influence the size of the basin of attraction for the labor equilibrium point and alter the stability of the equilibrium points. A tipping point exists in our model, where the quiescent equilibrium point transitions from stable to unstable and the phase space becomes completely biased toward the laboring equilibrium point. This change in stability is called a *bifurcation* and linear stability analysis allows us to compute the exact parameter values which will cause the stability to change. The end result of this analysis is a quantitative prediction for the *tipping point of labor*, the conditions under which the myometrial cell is permanently in the laboring phenotype leading to uterine emptying.

The point (0,0) corresponds to a myometrial cell that is not expressing any PR-B and has no inflammation. The solution (1,0) is the quiescent equilibrium corresponding to a physiological state where PR-B is at its maximal level with no inflammation. The solution (0,1) is the laboring equilibrium where inflammation is maximized and no PR-B is present. The last solution corresponds to an intermediate point between quiescent and laboring where the myometrial cell can pass into either phenotype.

In order to characterize the stability of these equilibrium solutions, we need to solve for the eigenvalues, *λ*_1_ and *λ*_2_, of the model at each equilibrium point. The sign of the eigenvalues, positive or negative, determines the stability and the formula for each eigenvalue tells us whether the eigenvalues can ever change sign. The eigenvalues are found by solving the characteristic polynomial equation of the Jacobian matrix, $\mathcal J$. By applying the quadratic formula, we can obtain an expression for both *λ*_1_ and *λ*_2_

6 $$\begin{array}{@{}rcl@{}} \lambda_{1} = \frac{-\beta + \sqrt{\left(\beta^{2} - 4\gamma\right)}}{2}\quad\lambda_{2} = \frac{-\beta - \sqrt{\left(\beta^{2} - 4\gamma\right)}}{2} \end{array} $$

where *β*=2(*bB*^∗^+*iI*^∗^)+*kB*^∗^+*I*^∗^−(*i*+*b*) and *γ*=*ib*(1−2*I*^∗^−2*B*^∗^+4*B*^∗^*I*^∗^)+*bB*^∗^(−*k*+2*kB*^∗^)+*iI*^∗^(2*I*^∗^−1)+*I*^∗^*B*^∗^(*k*−1). More details on the derivation of the eigenvalues can be found in the Appendix of derrivations.

The sign of *λ*_1_ and *λ*_2_ determine the type and stability of each equilibrium point (Table 1). The trivial equilibrium, (0,0), has two positive, real eigenvalues for all values of *b* and *i*, indicating it is always unstable and can be classified as a *source node*. Physiologically, a myometrial cell in this state is not exposed to inflammatory stimuli and is not expressing PR-B. This state cannot endure long and like the equilibrium point is unstable. The quiescent and laboring equilibria have two real, negative eigenvalues each and are thus stable *sink nodes*. Both of these are stable so long as both *b*<1 and *i*<1. The intermediate equilibrium point allows us to identify the tipping point as *i* and *b* change. This equilibrium has two real eigenvalues, one positive and one negative, thus the intermediate equilibrium is a semi-stable *saddle node*.

**Table 1:** Equilibrium solution stability conditions

Equilibrium	Trivial: (0,0)	Quiescent: (1,0)	Laboring: (0,1)	Intermediate: $\left (\frac {i(1-b)}{k-ib},\frac {b(k-i)}{k-ib}\right)$
Eigenvalues (*λ*_1_, *λ*_2_)	(*b,i*)	(*i*−*k*,−*b*)	(*b*−1,−*i*)	$\left (\frac {-\beta + \sqrt {(\beta ^{2} - 4\gamma)}}{2}, \frac {-\beta - \sqrt {(\beta ^{2} - 4\gamma)}}{2}\right)$
Stability	Unstable	Stable	Stable	Unstable
Condition	*b*>0 and *i*>0	(*i*<*k,b*>0)	(*b*<1,*i*>0)	*b*>0 and *i*>0

**Fig. 3:**
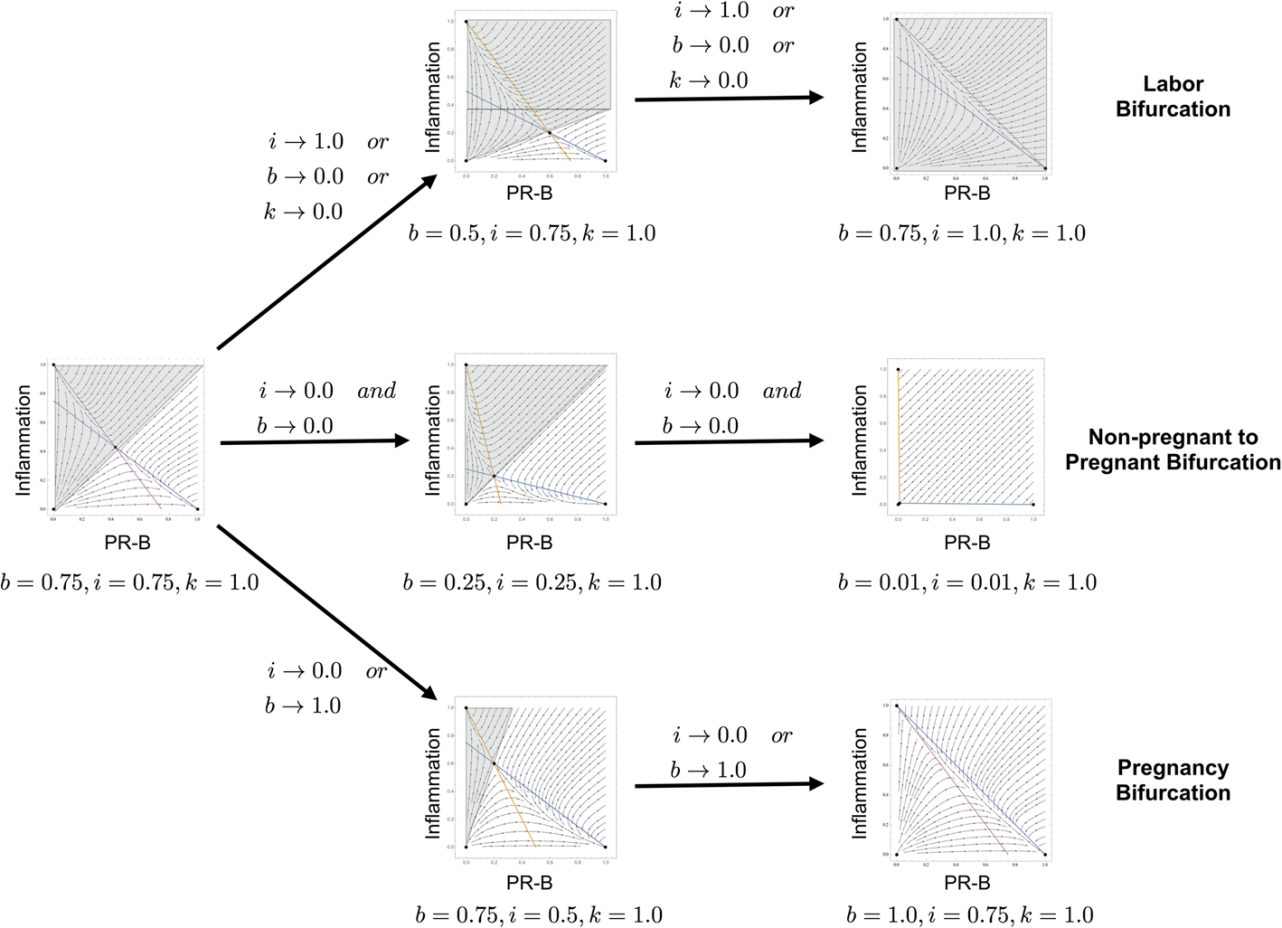
Phase Space Bifurcation Dynamics. Simulations of the three possible bifurcations in the PR-B/inflammation model The pro-labor bifurcation occurs as *i* approaches 1, or *b* approaches 0, or *k* approaches 0. The non pregnant to pregnant bifurcation occurs as *b* and *i* simultaneously approach 0. The pro-pregnancy bifurcation occurs as *i* approaches 0 or *b* approaches 1

The formula for the eigenvalues of the intermediate equilibrium indicates that three *bifurcations* are possible as *i* and *b* change. Firstly, if *i*=1 and *b*≤1 or if *k*≤*i*, then the quiescent equilibrium has one negative and one zero eigenvalue. In this case, the intermediate equilibrium point has moved through the phase space to collide with the quiescent equilibrium (Fig. 3). When these two equilibrium points combine, the quiescent equilibrium point changes from stable, where all temporal trajectories in the neighborhood of the equilibrium pointing toward it, to unstable with all the trajectories pointing away from the equilibrium point. This first bifurcation, the collision of the intermediate equilibrium with the quiescent equilibrium, corresponds to the physiological condition when the myometrium moves from quiescent to laboring. This condition corresponds to a probability of labor equal to one.

The converse of this is the second bifurcation that occurs when the intermediate equilibrium point collides with the laboring equilibrium point and the probability of labor is zero. This occurs when *b*=1 and *i*≤1, and results in a bifurcation where the laboring equilibrium point changes from stable to unstable. Physiologically this could correspond to a therapeutic intervention that preserves quiescence and prevents labor. Simulating therapeutic modulation of biomarkers that determine *b* and *i* could provide insight into what genes to modulate and what effect size is required to preserve quiescence.

When *k*=*bi*, the intermediate equilibrium becomes a singularity and is non-physiological. The third bifurcation occurs when *b* and *i* are both zero and the intermediate equilibrium collides with the trivial equilibrium point (Fig. 3). This third bifurcation may have physiological significance in the transition of the myometrial cell from non-pregnant to pregnant as inflammation and PR-B transition from inactive to active in the pregnancy uterus. However, since our model is based upon the activity of PR-B and inflammatory drivers during pregnancy, this bifurcation, though interesting, is beyond the scope of the present investigation.

The dynamical systems model was designed to explore the functional interaction between the anti-inflammatory actions of progesterone mediated by PR-B and the effect of inflammatory load on the contractile state of the human pregnancy uterus. In addition we sought to identify the conditions that induce a bifurcation in the model similar to the one that occurs when the uterus transitions to the laboring state. The dimensionless version of our model simplifies this task by enabling us to identify how changes in the three dimensionless parameters, *b*, *i*, and *k*, influence the trajectory of a hypothetical pregnancy phase space. The underlying rationale was that bifurcations correspond to physiologically important events in the timeline of pregnancy representing uterine quiescence and its transition to the laboring state. The interaction between the dimensionless model parameters *i* and *k* appears to be the most significant for initiating the labor bifurcation. The meaning of this interaction is that as long as the repressive capacity of PR-B, *k*, is greater than the activation rate of inflammation, *i*, quiescence will be maintained. This finding supports the PR-A:PR-B hypothesis since it recapitulates the important role of PR-B-mediated anti-inflammatory activity and shows how interference of this function, possibly by PR-A, destabilizes quiescence in favor of labor. Though we fixed *k* in the present analysis, we hypothesize that the level of *k* relative to *i* and *b* may be reflective of the trans-repressive activity of PR-A on PR-B. The phase space dynamics of modulating *k* seem to support this with higher values of *k* producing lower probabilities of labor and may be an interesting avenue for further investigation.

### Predictive modeling of parturition datasets

We next applied the dimensionless version of our model with *k*=1 in Eq. 3 to predict the onset of labor in a cohort of 18 patient samples from myometrial transcriptome studies. Given a value of (*b,i*) for a particular patient, the model’s phase space reflects the state of the pregnancy myometrium for that particular patient. Interpreting the phase space as a probability means that we have a metric for predicting the likelihood of the patient going into labor. It is now possible to test the predictive power of particular sets of biomarkers for predicting the laboring phenotype by assigning the values of *b* and *i* based on the values of molecular markers of PR-B activity and inflammation. We can also assess the gain in robustness and predictive power of labor classifiers by comparing the performance of our model’s 105,840 possible classifiers gainst the 264,600 possible one and two-gene null classifiers built with the normalized gene expression data.

In the case of the single gene null classifiers, the inflammatory surrogates all performed very well with average F-scores above 0.70 (Table 2). However, in the case of IL-6 only 61 % of the combinations of training samples could successfully build a classifier and the best performing classifier IL-1 *β* only had a 30 % success rate for classifier construction. The two-gene null classifiers had much higher success rates for construction classifiers, but rarely performed better than a random chance since most F-scores were around 0.50 (Table 2). In the case of building classifiers from gene expression data alone, the single gene models generally outperform the two-gene models, but the two-gene models are more robust against choice of training samples for building a classifier.

**Table 2:** Performance of of the null classifiers

Predictor	Average F-score	Classifier success rate
IL-6	0.74	0.61
IL-8	0.80	0.04
IL-1 *β*	0.89	0.30
FKBP5	0.46	0.14
FOXO1A	0.12	0.03
IL-6-IL-8	0.39	0.74
IL-6-IL-1 *β*	0.52	0.91
IL-6-FKBP5	0.59	0.92
IL-6-FOXO1A	0.52	0.91
IL-8-IL-1 *β*	0.36	0.58
IL-8-FKBP5	0.48	0.94
IL-8-FOXO1A	0.52	0.94
IL-1 *β*-FKBP5	0.49	0.87
IL-1 *β*-FOXO1A	0.51	0.91
FKBP5-FOXO1A	0.42	0.90

We see stronger classifier performance and success rates when the gene expression data is considered in the context of our dynamical systems model using the probability of labor. All classifiers built with the inflammatory surrogate genes IL-6 and IL- 1*β* had a 100 % success rate in classifier construction (Table 3). Further, these classifiers all had average F-scores above 0.77 with the combination of FKBP5 and IL-1 *β* begin the strongest. The model’s IL-6 and IL-1 *β* classifiers outperformed all two-gene null classifiers in both average F-score and CSR. Though the IL-8 and IL-1 *β* single gene null classifiers have higher average F-scores than our model classifiers, the low proportion of successful classifiers for both genes shows that these classifiers are extremely sensitive to changes in the training sample set. As such, the single gene classifiers are unreliable as biomarkers for labor.

**Table 3:** Performance of of the model classifiers

Predictor	Average F-score	Classifier success rate
IL-6-FKBP5	0.83	1.00
IL-6-FOXO1A	0.77	1.00
IL-8-FKBP5	0.63	0.14
IL-8-FOXO1A	0.68	0.27
IL-1 *β*-FKBP5	0.84	1.00
IL-1 *β*-FOXO1A	0.82	1.00

Identifying the specific inflammatory drivers that induce labor is an important step in identifying upstream and downstream therapeutic targets to delay the onset of premature labor. Interestingly, each of the inflammatory surrogate genes we used to construct our model’s classifiers has a subtly different biological function in the pregnancy uterus. IL-8 functions as a chemokine, drawing neutrophils and macrophages to tissues where it is expressed [32]. The lack of phenotypic predictability by IL-8, even when paired with a PR-B surrogate, may suggest that IL-8 does not play an important role in the inflammatory process of labor. In contrast IL- 1*β* and IL-6 [32] performed well as classifiers in our model when paired with PR-B surrogates, suggesting that the inflammatory processes associated with these genes are more important for labor onset than those of IL-8. In particular, IL-6 is a cytokine that plays an important role in both the canonical and non canonical JAK-STAT signaling pathway [33, 34], a pathway that may integrate effects of circulating and local myometrial cytokines. Recent work examining IL-6 as a blood-based biomarker for labor [35] provides further evidence that understanding and modeling this cytokine in the myometrium could be key to elucidating the driver pathways of labor.

The small sample sizes in the transcriptome datasets (*N*=18) cause us to exercise caution in the data analysis. The nonparametric methods of constructing confidence intervals and testing the separation of the NIL and IL groups are less powerful than comparable parametric testing, but were ultimately more appropriate due to their resistance to outliers, non normality of the data, and small sample size. Our approach of bootstrapping a distribution of classifiers and performance metrics allowed us to overcome the limitations of our cohort size and systematically test 370,440 classifiers and assess the performance of our model in a variety of contexts.

The present investigation of the predictive power of our model was limited by the availability of in vivo temporal data for the activity of PR-B and inflammation in the context of the pregnancy myometrium. If there were bloodborne biomarkers that could be used to infer the activity of these quantities, then it would be possible to test the application of our model in a clinical context modeling and predicting the pregnancy trajectories of actual women by measuring two biomarkers, one for PR-B and one for inflammation. This work is the first step in that direction by using transcriptome data from the myometrium to train a predictive model and demonstrate that our model provides the mechanistic hypothesis and framework to increase the predictive power of gene expression data.

## Conclusions

This mathematical model of the PR-A:PR-B hypothesis of human parturition produces qualitative dynamics which mimic those observed in vitro and in vivo. A novel interpretation of the phase space of a dynamical system as a probability space enables predictive modeling of all possible phenotypic states of the pregnancy myometrium in a patient specific manner. Predictive modeling of patient datasets shows that our model makes accurate predictions of the laboring phenotype in patients, performing best when the PR-B surrogate FKBP5 and inflammatory surrogate IL-1 *β* are used to fit the dimensionless model. Linear stability analysis shows three phenotypically interesting phase space bifurcations exist in our model and provides a quantitative tipping point for the myometrium transitioning to the contractile phenotype given our model. This dynamical systems model of progesterone receptor interactions in the pregnancy myometrium provides a plausible explanation for the observed biochemical dynamics in the literature and is a first step in more sophisticated modeling of human partition with dynamical systems models. Our model provides a framework where if a woman’s PR-B and inflammatory activity can be determined from blood-based biomarkers, then we can produce patient specific trajectories characterizing a woman’s likelihood of labor and the variables to modulate to prevent PTB.

## Appendix of derrivations

### Model nondimensionalization and simplification

There are six parameters in our model ${k_{1}, k_{2}, B_{c}, I_{c}, \hat {i}, \hat {b}}$ with various units. Nondimentionalization is a tool for simplifying our model whereby these parameters are replaced with dimensionless constants. While nondimensionalization can make it difficult to pinpoint the influence of individual parameters on the system’s behavior, this concern is minimal since we only have six parameters in our model. Unpacking the influence of particular dimensionless constants and the parameters that constitute those constants is thus straightforward for our model. The units for $\hat {I}$, $\hat {B}$, *B*_*c*_, and *I*_*c*_ are the amount of PR-B or inflammation present, similar to a concentration. Time *t* is given in weeks. The rate constants $\hat {I}$ and $\hat {B}$ are in units $\frac {1}{\text {weeks}}$ while the rate constants *k*_1_ and *k*_2_ are in units $\frac {1}{\text {concentration} * \text {weeks}}$. We define three dimensionless variables for our model,

7 $$\begin{array}{@{}rcl@{}} B = \frac{\hat{B}}{B_{c}} \quad \quad I = \frac{\hat{I}}{I_{c}} \quad \quad \tau = tk_{1}I_{c} \end{array} $$

Substituting these for $\hat {B}$, $\hat {I}$, and *t* makes the PR-B equation,

8 $$\begin{array}{@{}rcl@{}} B_{c}I_{c}k_{1}\frac{dB}{d\tau} = \hat{b}B_{c}B(1-B)-k_{1}B_{c}I_{c}BI, \end{array} $$

and the inflammation equation,

9 $$\begin{array}{@{}rcl@{}} {I_{c}^{2}}k_{1}\frac{dI}{d\tau} = \hat{i}I_{c}I(1-I)-k_{2}B_{c}I_{c}BI. \end{array} $$

We simplify the equations by dividing by *B*_*c*_*I*_*c*_*k*_1_ in the equation for $\frac {dB}{d\tau }$ and by ${I_{c}^{2}}k_{1}$ in the equation for $\frac {dI}{d\tau }$. The result is the dimensionless model,

10 $$\begin{array}{@{}rcl@{}} \frac{dB}{d\tau} &=& \frac{\hat{b}}{k_{1}I_{c}}B(1-B)-BI, \\ \frac{dI}{d\tau} &=& \frac{\hat{i}}{k_{1}I_{c}}I(1-I)-\frac{k_{2}B_{c}}{k_{1}I_{c}}BI. \end{array} $$

We can now define three dimensionless constants, $R_{1} = \frac {\hat {b}}{k_{1}I_{c}}$, $R_{2} = \frac {\hat {i}}{k_{1}I_{c}}$, and $R_{3} = \frac {k_{2}B_{c}}{k_{1}I_{c}}$. Substituting these yields the final version of the dimensionless model,

11 $$\begin{array}{@{}rcl@{}} \frac{dB}{d\tau} = R_{1}B(1-B)-BI,\\ \frac{dI}{d\tau} = R_{2}I(1-I)-R_{3}BI. \end{array} $$

We infer the activity of PR-B and pro-inflammatory drivers with two PR-B responsive genes to serve as surrogates for PR-B, FOXO1A and FKBP5, and three pro-inflammatory genes to serve as surrogates for inflammation, IL- 1*β*, IL-6 and IL-8. The normalized values of the data for these genes *N*_*j*_, are calculated using the equation,

12 $$ N_{j} = \frac{G_{j}- m}{M-m}  $$

where *G*_*j*_ is the value of the gene for patient *j*, *M* is the maximum expression value for that gene across patients in the dataset, and *m* is the minimum value of that gene across patients in the dataset. One consequence of this normalization procedure is that the transcriptome data has been nondimensionalized. Therefore, the dimensionless constants and dimensionless trascriptome data can be seamlessly combined so that the surrogate genes parameterize the dimensionless model for each patient. This equation bounds the values for the PR-B and inflammation surrogates from 0 to 1 and makes the natural choice of values for critical levels of PR-B and inflammation *B*_*c*_=*I*_*c*_=1. The dimensionless parameters then become,

13 $$\begin{array}{@{}rcl@{}} R_{1} = \frac{\hat{b}}{k_{1}} = b \quad \quad R_{2} = \frac{\hat{i}}{k_{1}} = i \quad \quad R_{3} = \frac{k_{2}}{k_{1}} = k. \end{array} $$

Now our model can be rewritten as,

14 $$\begin{array}{@{}rcl@{}} \frac{dB}{d\tau} = bB(1-B)-BI, \quad \quad \frac{dI}{d\tau} = iI(1-I)-kBI, \end{array} $$

where the values of *b* and *i* are determined by the normalized dimensionless values for the PR-B and inflammatory surrogate genes respectively and the value of *k* corresponds to the strength of PR-B’s anti-inflammatory actions.

### Calculating the probability of labor for each patient

Next we quantify the behavior of *k* in order to apply our model to patient data. To do this, we have to derive the steady states solutions for our model. These solutions are the values of *B* and *I* which cause $\frac {dB}{d\tau }=\frac {dI}{d\tau }=0$ and correspond to a state where the system undergoes no change. There are three steady states, *equilibrium points*, which are easy to derive. These occur when the ordered pair for PR-B and inflammation, (*B,I*), is equal to,

15 $$\begin{array}{@{}rcl@{}} (0, 0) \quad \quad \quad \quad (1, 0) \quad \quad \quad \quad (0, 1) \end{array} $$

where (0,0) is the trivial equilibrium point where neither PR-B nor inflammation is present, (1,0) is the *quiescent equilibrium* where PR-B is maximal and there is no inflammation, and (0,1) is the *laboring equilibrium* where there is no PR-B and inflammation is maximal. The quiescent equilibrium correspond to a PR-B dominant state and laboring equilibrium corresponds to an inflammatory dominant state. There is a fourth equilibrium point which exists for some values of *b* and *i* between the quiescent and laboring equilibrium which we will designate as the *intermediate equilibrium*, (*B*^∗^,*I*^∗^). We obtain this equilibrium point by first setting our model equations equal to zero,

16 $$\begin{array}{@{}rcl@{}} \frac{dB}{d\tau} =0= bB^{*}(1-B^{*})-B^{*}I^{*},\\ \frac{dI}{d\tau} =0= iI^{*}(1-I^{*})-kB^{*}I*, \end{array} $$

and

17 $$\begin{array}{@{}rcl@{}} \frac{dI}{d\tau} =0= iI^{*}(1-I^{*})-kB^{*}I*. \end{array} $$

Simplifying this becomes,

18 $$\begin{array}{@{}rcl@{}} 0= b(1-B^{*})-I^{*}, \quad \quad 0= i(1-I^{*})-kB^{*}. \end{array} $$

This results in two equations, one for *B*^∗^ and one for *I*^∗^,

19 $$\begin{array}{@{}rcl@{}} I^{*} = b-bB^{*}, \quad \quad B^{*} = \frac{i}{k}(1-I^{*}). \end{array} $$

Depending on how we chose to perform the substitution, *B*^∗^ into the equation for *I*^∗^ or *I*^∗^ into the equation for *B*^∗^, we can derive two forms of the same intermediate equilibrium point. These are,

20 $$\begin{array}{@{}rcl@{}} (B^{*},I^{*}) &=& \left(\frac{i(1-b)}{k-ib},\frac{b(k-i)}{k-ib}\right)\\&=&\left(\frac{i^{2}b(1-k)-ik(1-b)}{k(bi-k)}, \frac{kb(i-1)}{bi-k}\right). \end{array} $$

Since the values of *b* and *i* are determined by PR-B and inflammatory surrogate genes scaled from 0 to 1, these terms are bounded to that interval. Furthermore, since *B* and *I* are bounded by *B*_*c*_=1 and *I*_*c*_=1, *B*^∗^ and *I*^∗^ are bounded to the square domain with vertices (0,0), (0,1), (1,1), and (1,0) and area 1. So, the intermediate equilibrium point, in both forms, should satisfy the constraints 0≤*B*^∗^≤1 and 0≤*I*^∗^≤1. By considering how this constraint impacts both forms of the intermediate equilibrium we can derive a set of constraints for the values of *k*. In order to allow for the full range of values of *i*, we find that *i* and *k* satisfy 0≤*i*<*k*≤1.

In the limit in the case where *i*=*k*, the intermediate equilibrium point equals the quiescent equilibrium point (1,0). If we visualize this state in *phase space* we see that all the vectors point away from the quiescent equilibrium point toward the laboring equilibrium. In phase space, these vectors define *trajectories* that indicate how the system would evolve in time given a certain starting point. The set of vectors pointing toward the laboring equilibrium point is known as the *basin of attraction* for the laboring equilibrium point. We compute a *probability of labor* equal to the area of the laboring equilibrium point’s basin of attraction divided by the area of the domain, which in our case is 1. The area of the basin changes as *k*, *i*, and *b* change. This probabilistic interpretation of a phase space is reasonable under the assumptions that all possible pairs of values of *B* and *I* in the domain occur with equal likelihood. For example, in the case where *k*=*i* and *b* is fixed at 0.5, the probability of labor is equal to 1, the entire domain is the basin of attraction for the laboring equilibrium, which means that quiescence is impossible. In order to ensure that quiescence is a possibility, we set *k*=1 so that only one value of *i*, *i*=1, results in a probability of labor equal to 1 enabling us to explore the full range of values for *b* and *i*. Thus, the model we apply to patient data is

21 $$\begin{array}{@{}rcl@{}} \frac{dB}{d\tau} = bB(1-B)-BI, \quad \quad \frac{dI}{d\tau} = iI(1-I)-BI. \end{array} $$

### Characterizing the stability of the quiescent and laboring equilibria

We analyzed the dimensionless form of our model and computed the eigenvalues for the four equilibrium points,

22 $$\begin{array}{@{}rcl@{}} (0, 0) \quad (1, 0) \quad (0, 1) \quad \left(\frac{i(1-b)}{k-ib},\frac{b(k-i)}{k-ib}\right) \end{array} $$

where (0,0) corresponds to a myometrial cell that is not expressing any PR-B and has no inflammation. The solution (1,0) corresponds to a myometrial cell that does not express PR-B and has no inflammation. The solution (1,0) is the quiescent equilibrium corresponding to a physiological state where PR-B is at its maximal level with no inflammation. The solution (0,1) is the laboring equilibrium where inflammation is maximized and no PR-B is present. The last solution corresponds to an intermediate point between quiescent and laboring where the myometrial cell can pass into either phenotype.

In order to characterize the stability of these solutions, we need to solve for the eigenvalues of the model *λ*_1_ and *λ*_2_ at each equilibrium point from (5). The sign of the eigenvalues, positive or negative, determines the stability and the formula for each eigenvalue tells us whether the eigenvalues can ever change sign. This is done by solving the characteristic polynomial equation of the Jacobian matrix, $\mathcal {J}$. We begin by computing $\mathcal {J}$ for our model at a general equilibrium point (*B*^∗^,*I*^∗^),

23 $$\begin{array}{@{}rcl@{}}  \mathcal{J}\! = \!\left(\!\begin{array}{cc} \frac{dB'}{dB} & \frac{dB'}{dI} \\ \frac{dI'}{dB} & \frac{dI'}{dI}\\ \end{array}\!\right)\! =\! \left(\!\begin{array}{cc} b - 2bB^{*}-I^{*} & -B^{*}\\ -I^{*} & i-2iI^{*} - kB^{*}\\ \end{array}\!\right). \end{array} $$

The characteristic polynomial can be obtained by taking the determinant of the matrix,

24 $$\begin{array}{@{}rcl@{}}  \left(\!\lambda I \,-\, {\mathcal{J}}\right)\,=\, \left(\!\!\begin{array}{cc} \lambda - b + 2bB^{*}+I^{*} & -B^{*}\\ -I^{*} & \lambda - i+2iI^{*} + kB^{*}\\ \end{array}\!\!\right). \end{array} $$

Setting this determinant to zero gives us the eigenvalues, the roots of the characteristic polynomial whose signs determine the stability of the equilibrium solutions:

25 $$ 0 = \lambda^{2} + \lambda\beta+ \gamma.  $$

where

26 $$\begin{array}{@{}rcl@{}} {}\beta = 2\left(bB^{*} + iI^{*}\right) + kB^{*} + I^{*} -(i +b) \end{array} $$

and

27 $$ \begin{aligned} \gamma =&\; ib\left(1-2I^{*}-2B^{*}+4B^{*}I^{*}\right)+bB^{*}\left(-k+2kB^{*}\right)\\ &+iI^{*}\left(2I^{*}-1\right) + I^{*}B^{*}(k-1) \end{aligned}  $$

By applying the quadratic formula, we can obtain an expression for both *λ*_1_

28 $$\begin{array}{@{}rcl@{}} \lambda_{1} = \frac{-\beta + \sqrt{(\beta^{2} - 4\gamma)}}{2} \end{array} $$

and *λ*_2_

29 $$\begin{array}{@{}rcl@{}} & &  \lambda_{2} = \frac{-\beta - \sqrt{(\beta^{2} - 4\gamma)}}{2} \end{array} $$

The sign of *λ*_1_ and *λ*_2_ determine the type and stability of each equilibrium point. The trivial equilibrium, (0,0), has two positive, real eigenvalues for all values of *b* and *i* indicating it is always unstable and can be classified as a *source node*. Physiologically, a myometrial cell in this state is not exposed to inflammatory stimuli and is not expressing PR-B. This state cannot endure long and like the equilibrium point is unstable, all trajectories are pointing away from the equilibrium. The quiescent and laboring equilibria have two real, negative eigenvalues each and are thus stable *sink nodes*. Both of these are stable so long as both *b*<1 and *i*<1. The intermediate equilibrium point allows us to identify the tipping point as *i* and *b* change. This equilibrium has two real eigenvalues, one positive and one negative, thus the intermediate equilibrium is a semi-stable, *saddle node*.

The formula for the eigenvalues of the intermediate equilibrium indicates that three *bifurcations* are possible as *i* and *b* change. Firstly, if *i*=1 and *b*≤1 or if *k*≤*i*, then the quiescent equilibrium has one negative and one zero eigenvalue. In this case, the intermediate equilibrium point has moved through the phase space to collide with the quiescent equilibrium. When these two equilibrium points combine, the quiescent equilibrium point changes stability from stable, where all temporal trajectories in the neighborhood of the equilibrium pointing toward it, to unstable with all the trajectories pointing away from the equilibrium point. Similarly, if *b*=1 and *i*≤1 the intermediate equilibrium collides with the laboring equilibrium resulting in a bifurcation where the laboring equilibrium point changes from stable to unstable. When *k*=*bi* the intermediate equilibrium becomes a singularity and is non-physiological. The third bifurcation occurs when *b* and *i* equal 0 and the intermediate equilibrium collides with the trivial equilibrium point.
